# Clinical aspects of human rabies in the state of Ceará, Brazil: an overview of 63 cases

**DOI:** 10.1590/0037-8682-0509-2021

**Published:** 2021-12-17

**Authors:** Naylê Francelino Holanda Duarte, Roberto da Justa Pires, Victoria Forte Viana, Levi Ximenes Feijão, Carlos Henrique Alencar, Jorg Heukelbach

**Affiliations:** 1 Universidade Federal do Ceará, Faculdade de Medicina, Programa de Pós-Graduação em Saúde Pública, Fortaleza, CE, Brasil.; 2 Universidade de Fortaleza, Centro de Ciências da Saúde, Fortaleza, CE, Brasil.; 3 Secretaria da Saúde do Estado do Ceará, Fortaleza, CE, Brasil.

Dear Editor:

We completely agree with the reader’s comments and share the same perspective. In the case of category III exposure, administration of rabies immunoglobulin is formally indicated, and patients undergoing post-exposure prophylaxis should be monitored until the entire regimen is completed. Unfortunately, of the 63 patients with rabies occurring over 50 years, as described in our paper, only 14 (22%) sought medical care before the onset of symptoms, and most patients did not receive the post-exposure prophylaxis regimen, according to the World Health Organization (WHO) guidelines; several patients failed to complete the treatment regimen. This highlights challenges faced by the population in accessing the health system, especially in rural areas, and insufficient knowledge among the population and healthcare professionals, mainly from the 1970s to the 1990s. Although there has been substantial improvement in precise clinical management over the past years, there is an ongoing need for continuous medical education, considering that several healthcare professionals remain unfamiliar with WHO guidelines regarding post-exposure prophylaxis^1^.
